# Role of Haematological Parameters as an Indicator of Acute Malarial Infection in Uttarakhand State of India

**DOI:** 10.4084/MJHID.2013.009

**Published:** 2013-01-02

**Authors:** Smita Chandra, Harish Chandra

**Affiliations:** Department of Pathology, Himalayan Institute of Medical Sciences. Swami Ram Nagar, Doiwala, Dehradun-248140, Uttarakhand, India

## Abstract

**Background:**

Malaria may be associated with complications which may be avoided by early diagnosis and treatment. Microscopic diagnosis showing presence of malarial parasites is needed for confirmation which at times may be unreliable and requires technical expertise. The present study was conducted to statistically analyze the haematological parameters including platelet indices which can give initial hint for malarial infection and therefore prompt the laboratory physician for active search of the parasite microscopically.

**Methods:**

A retrospective study was conducted which included 334 cases of acute malaria caused by Plasmodium vivax, falciparum and dual infection along with 100 cases of control. Routine haematological parameters along with platelet indices (MPV and PDW) which are easily available on automated cell counter were statistically analyzed to assess their role as indicators for malaria.

**Results:**

Leukocyte count and platelet count were significantly lower in cases of acute malaria in comparison to controls (p value <0.001). Platelet count<150×109/l showed 87.2% sensitivity, 65% specificity, 89.3% positive predictive value and 2.49 likelihood ratio for the infection. PDW of 6–10 and MPV>8 fl had 71.9% and 61.5% sensitivity and 78.2% and 77.7% positive predictive value respectively for infection. Platelet count <150×109/l and MPV>8 fl was comparatively more sensitive indicator for vivax (88% and 70.8% respectively) than falciparum (84.8% and 50.4% respectively) and PDW 6–10 was more sensitive indicator for falciparum (82.6%) than vivax (69.5%) infection.

**Conclusion:**

Thrombocytopenia (<150×109/l) and low leukocyte count (<4×109/l) may be used as probable indicator for malaria in endemic countries. Higher MPV (>8 fl) and PDW of 6–10 also show considerable sensitivity for malarial infection. In addition, thrombocytopenia (<150×109/l) and higher MPV (>8 fl) was more sensitive for vivax infection while PDW 6–10 was more sensitive for falciparum infection.

## Introduction

Malaria is an important infectious protozoan disease and it has been estimated that worldwide there are 300–500 million cases of malaria per year and 1.5 – 2.7 million deaths due to it.^1^ Although it has been considered to be eliminated in United States, Canada, Europe and Russia but local transmission has been reported in these areas due to imported cases.^2^ The early diagnosis of malaria is the key feature for its prompt treatment and prevention of complications which may include coma, hypoglycaemia, acidosis, renal failure or pulmonary oedema Microscopic diagnosis is needed for confirmation of malaria but it requires technical expertise and at times may be unreliable when poorly executed.^3^ Certain haematological changes which include low platelet count, haemoglobin concentration and hematocrit have been reported to be associated with malaria.^4^,^5^ Recently studies have also been conducted to analyze the role of platelet indices to discriminate various causes of thrombocytopenia including malaria.^6^

The study was therefore conducted to statistically analyze all the haematological parameters including platelet indices that are easily available on blood count autoanalyzer and can give an initial hint for acute malarial infection caused by various species of Plasmodium. The parameters indicating towards malarial infection will in turn prompt for vigilant search of the parasite on peripheral blood smear and will thus avoid missing of cases of malaria.

## Material and Methods

The present study was conducted retrospectively over period of two years from August 2008 till August 2010 in the haematology laboratory of the institute situated in the Uttarakhand state of India which includes Himalayan and sub Himalayan region of north India. The study included randomly selected cases of acute malaria which presented with fever for within a week caused either by Plasmodium *falciparum* (PF), *vivax* (PV) or dual infection (PV+PF) that were retrieved from the archives of the laboratory. Detailed clinical history and prior medication were noted for every case where ever possible. The cases which gave clear history of receiving antimalarials before being referred to the lab were excluded from the study. All the cases were confirmed to be caused by Plasmodium by demonstration of the trophozoite or gametocyte of the parasite by microscopic examination of the peripheral blood smear. The cases were also confirmed by using rapid immunochromatographic card test for detection of PV, PF or PV+PF infection on whole blood (QDx Malaria Pv/Pf malaria card test, Piramal Healthcare Limited, Mumbai, India) whenever required. Routine haematological parameters which included haemoglobin level (Hb), total leukocyte count (TLC), red blood cell count (RBC), mean cell volume (MCV), red cell distribution width (RDW), platelet count (PC), mean platelet volume (MPV) and platelet distribution width (PDW) which were available on automated analyzer were recorded for every patient. These haematological parameters were obtained by subjecting the blood samples of the patients which were collected in ethylene diamine tetra acetic acid (EDTA) anticoagulant monovette tubes to MS-9 Automatic Full Digital Cell Counter (Melet Schloesing Laboratories, Cergy Pontoise, France). This automated analyzer is a three part differential analyzer and works on the principle of impedance method. 100 patients were randomly taken as controls which were also retrieved from the archives of the same lab during the same period of time and presenting with acute febrile illness for within a week. Their detailed history showed no prior medication or treatment before presenting to the laboratory. These controls were negative for malarial parasite on peripheral blood smear examination or rapid malarial test but were confirmed to be due to other causes such as typhoid by Widal test, bacterial infections by blood culture, dengue by viral antigen detection or immunoglobulins and other viral infections by Enzyme linked immunosorbent assay (ELISA). Statistical analysis was carried out by using SPSS version 15.0 for windows (Chicago, IL, USA). Results were expressed as means ± SD and student’s t test was applied to compare the parameters between cases and controls. P<0.001 was considered statistically significant. The sensitivity and specificity of relevant haematological parameters as an indicator of malarial infection were also assessed by performing receptor operating curve (ROC) analysis.

## Results

The study included 334 cases of malarial infection with mean age of 33.13 years and male female ratio of 1.63:1. Out of these 233 cases (69.8%) were positive for PV, 92 cases (27.5%) for PF and 9 cases (2.7%) showed dual infection by PV and PF. The controls showed mean age of 29.12 years with male female ratio of 1.1:1. It was observed that out of 334 cases of malarial infection 248 were present in the months of July to October. Table 1 shows the comparison of different characteristics between cases and controls. It shows that treatment history was available for 303 cases and out of these 275 patients gave history of no prior treatment before presenting to the laboratory while 28 had received treatment including antipyretics etc. but no antimalarials. 31 cases could not clearly elicit whether they have received any prior treatment or if received then which type of treatment received due to illiteracy. Table 2 shows the mean and standard deviation of the various haematological parameters and comparison of these parameters between cases and controls. It shows that TLC and PC were significantly lower in cases of malarial infection in comparison to controls. The comparison of the haematological parameters between PV, PF and dual infection with controls also showed similar results (p value <0.001 for TLC and PC). Table 3 shows that PC<150×10^9^/l showed 87.2% sensitivity, 65% specificity, 89.3% positive predictive value and 2.49 likelihood ratio for malarial infection. In addition, PDW in the range of 6–10 and MPV>8 fl had 71.9% and 61.5% sensitivity and 78.2% and 77.7% positive predictive value respectively for malarial infection. It was also observed that positive predictive value for malaria at PC<150×10^9^/l increased to 90.8% during the months of July to October when the cases of malaria increased. The positive predictive value for the infection at TLC<4×10^9^/l was also 81.1% during these months. Table 4 shows the statistical analysis of haematological parameters as an indicator of PV and PF separately.

**Figure 1:**
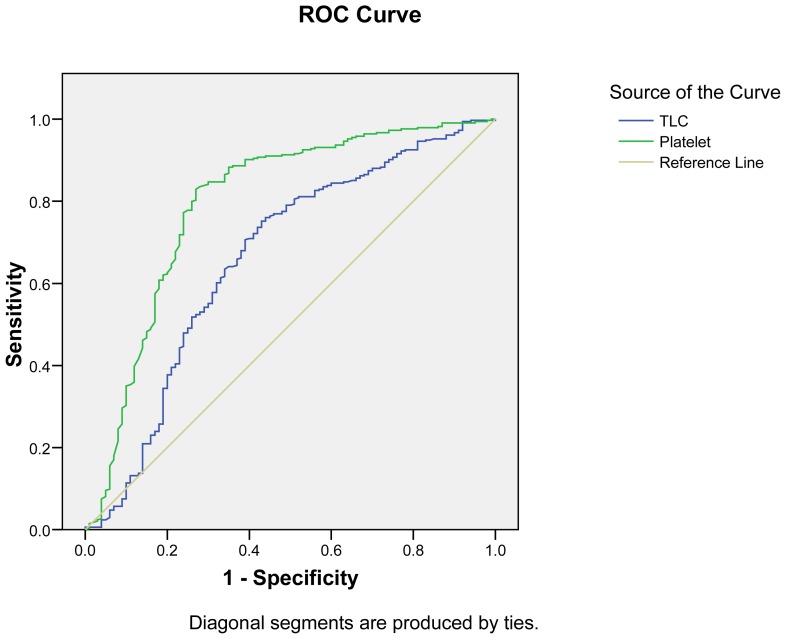
Receptor Operating Characteristic (ROC) curve of TLC (Total Leukocyte Count) and platelet count for malarial infection.

Table 4 shows that platelet count <150×10^9^/l was more sensitive (88% for PV and 84.8% for PF) while TLC < 4×10^9^/l was more specific (90%) for PV and PF infection. In addition, MPV>8 fl is comparatively more sensitive indicator for PV infection than for PF infection and PDW 6–10 is more sensitive indicator for PF infection. ROC curve analysis showed that cut off of TLC < 9.22×10^9^/l was 70.7% sensitive and 61% specific as an indicator for malarial infection while PC< 113.20×10^9^/l was 77.8% sensitive and 76% specific as indicator for malarial infection (Figure 1).

## Discussion

Malaria is considered a major public health problem in India and total malaria cases reported in 2008 were 1.52 million with Annual Parasite Incidence (API) of 1.40 and Slide Positivity Rate (SPR) of 1.60.^7^ Although India accounted for 66% of confirmed cases of malaria in South East Asia region in 2010 but there was reduction of 28% of cases between 2000 and 2010 and it is considered that India is in the control phase of malaria.^8^,^9^ Severe malariamay be associated with various complications such as hypoglycaemia, acidosis, coma and rarely septicaemia and prevention of these complications require early confirmed diagnosis followed by adequate antimalarian treatment. Peripheral blood smear examination is used fo laboratory confirmation of parasite but it requires technical expertise, repeated smear examination and thus may prove unreliable and wasteful when poorly executed.^3^,^10^ Clinical presentation of malaria is also variable and may overlap with symptoms of other bacterial and viral infections such as dengue, typhoid etc. and thus posing difficulty in diagnosis. Dengue is also widely prevalent in India and during 2009 about 15,509 cases were reported with 89 deaths while 1.03 million cases and 421 deaths were reported of typhoid in India in 2009.^11^,^12^ Therefore it is essential to discriminate between these infections so that early diagnosis of malaria is possible. Various haematological parameters which are easily available on automated cell counters may be useful in this regard. The association of thrombocytopenia and malaria have been reported by various studies. 13,14,15 In India, PV is widespread in the southern state of Tamil Nadu, PF in Orissa state while mixed infections are predominant in the west of the country.^16^,^17^ The present study showed that total leukocyte count and platelet count were significantly lower in cases of malaria in comparison to controls and thrombocytopenia was more sensitive and had higher positive predictive value and likelihood ratio for PV infection in comparison to PF (Table 4). Dual infection by PV and PF was not considered adequate for statistical analysis as it comprised of only 9 cases out of total 334 malarial cases. A study from Bikaner in northwestern India had similar observations as present study which concluded that association of thrombocytopenia was statistically more significant with PV monoinfection as compared to PF.^18^ Another study from north-east Papua also observed that platelet count was significantly lower in vivax malaria in comparison to falciparum malaria initially and mean platelet count increased with time after treatment.^19^ Studies have reported the significance of thrombocytopenia in relation to different species of Plasmodium and concluded that association of thrombocytopenia was statistically more significant with PV monoinfection as compared to PF and mean platelet count increased over time. 18,19 Saravu *et al* in their study from Karnataka, southern state of India have concluded that thrombocytopenia is observed in malaria and incidence is similar in vivax and falciparum infection and the patients with complications had significantly lower platelet count in comparison to patients without complications.^20^ In contrast, studies have also observed that thrombocytopenia less than 20×10^9^/L is statistically more common with falciparum infection.^21^ Another study also analyzed the clinico-hematological parameters during episode of acute malaria by PV in Uttarakhand region of India and observed that thrombocytopenia (<150×10^9^/l) was the commonest manifestation and associated with complicated malaria.^22^ Studies from Delhi, India have also observed thrombocytopenia in 80.37% to 96% cases of PV malarial infection and have challenged the use of terminology of “benign tertian malaria” for PV infection as thrombocytopenia with other complications are observed in high percentage in PV malarial infection patients.^23^,^24^ The authors therefore suggest that further studies in this region may also be done which correlate the presence of complications with thrombocytopenia in relation to different malarial species and different levels of platelet count. The pathogenesis of thrombocytopenia in malaria have been suggested due to peripheral destruction, splenic pooling or consumption coagulopathy but de Mast *et al* have hypothesized that thrombocytopenia in early malaria is associated with GPIb shedding in absence of systemic platelet activation and consumptive coagulopathy.^25^ Malaria is considered to show seasonal variation in India with maximum prevalence in rainy season from July to November.^7^ The study also showed that positive predictive value of thrombocytopenia for the malaria infection increased during the months of July to October when maximum number of cases were reported in the laboratory while it decreased during the other months and thus the role of thrombocytopenia as predictor for malaria is retained and shows increase when the incidence of malaria increases. Recently MPV has also been analyzed for various diseases including its role as discriminating guide for thrombocytopenia.^6^,^26^ Platelet indices have been rarely analyzed for malaria infection and the present study observed that MPV >8 fl and PDW in range of 6–10 showed significant sensitivity (61.5% and 71.9% respectively) for malarial infection but with low specificity (41% and 33% respectively) (Table 3). The study also observed that higher MPV (> 8 fl) was more sensitive indicator for PV infection while PDW 6–10 was more sensitive for PF infection (Table 4). Although studies have concluded that MPV was higher in malaria infected children than non malaria infected children but it has also been observed that malaria with thrombocytopenia involving bone marrow had lower MPV in comparison to cases without bone marrow involvement which showed higher MPV.^6^,^27^ Chandra et al have concluded that increased MPV was observed in those malaria patients in which the pathogenesis of thrombocytopenia was due to increased peripheral destruction (hyperdestuctive) while decreased MPV was present in those patients in which the parasite was observed in bone marrow indicating that thrombocytopenia was due to bone marrow disease (hypoproductive).^6^ However in the present study, although MPV was observed to be increased in malarial infections but the pathogenesis of thrombocytopenia including hyperdestructive or hypoproductive thrombocytopenia cannot be confirmed as bone marrow examination was not done in very few cases.

It was also observed in the present study that leukocyte count was significantly lower in cases than in comparison to controls and count <4×10^9^/l showed high specificity (90%) but low sensitivity (11.3%) for malarial infection (Table 2 and 3). This is in contrast to previous study which concluded that leukocyte count was not predictive for malaria infection.^10^ The present study also observed that other haematological parameters including Hb, RBC count MCV, RDW are inadequate indicators for malarial infection. It was observed in this study that out of total 334 cases of malaria 275 had received no prior treatment and 28 had received partial treatment but no antimalarial before their blood sample was analyzed in the laboratory (Table 1). This may exclude the possibility of bias due to antimalarial treatment to some extent but the study also showed that no treatment history was available for 31 cases. This was predominantly due to illiterate patients in which treatment history could not be elicited. Thus, an important limitation of the present study was that the patients may have got partial treatment before being referred to the institute and, therefore, causing variation in the haematological parameters resulting as confounding factor. Therefore, this study should be further extended in order to permit a statistical analyse of the changes in haematological parameters due to antimalarial treatment received. Further studies analysing the haematological parameters at 0 time, when no treatment has been given, and after the treatment should also be done. In fact few studies have reported the haematological parameters in malarial infection throughout the course of the disease, and in reference to the treatment; Tangpukdee et al.^28^ observed that leukocyte count was significantly very low initially (day 0) and returned to normal value by day 28. Another limitation of the studies is the possibility of bias in random selection of cases and controls specially related to seasonality along with concomitant febrile illness giving thrombocytopenia. In fact in our experience the predictive value of both thrombocytopenia and leucopenia changed throughout the year increasing to 90.8% during the months of July to October when the cases of malaria increased.

Therefore, our study concludes that thrombocytopenia (<150×10^9^/l) and low leukocyte count (<4×10^9^/l) may be used as probable indicator for malaria infection in endemic regions. A high MPV (>8 fl) and PDW in range of 6–10 also show considerable sensitivity for malarial infection. Thrombocytopenia is more significant indicator for PV infection than for PF infection. In addition, higher MPV (> 8fl) is also more sensitive indicator for PV infection while PDW 6–8 is more sensitive for PF infection. Therefore we suggest that if a patient, in a malarial endemic region, is presenting with acute febrile illness (<1 week) and the blood count analysis shows thrombocytopenia (<150×10^9^/l) with low leukocyte count (<4×10^9^/l) along with increased MPV (>8 fl), he should be suspected for malarial infection and a prompt search of the parasite should be made on peripheral blood smear in order to avoid missing the diagnosis. Although these haematologic parameters are only indicators of probable malarial infection, they are an inexpensive and easily available guide for active search of the parasite, in order to rapidly institute a specific treatment.

## Figures and Tables

**Table 1:** Comparison of characteristics between cases of malaria and controls.

Characteristics	Cases (n=334)	Controls (n=100)

Age (Mean)	33.13 years	29.12 years
Male Female ratio	1.63:1	1.1:1
History of fever	Within one week	Within one week
Treatment history available before blood sample analysis	303	100
*No treatment received*	275	100
*Partial treatment received for fever but no antimalarials*	28	
*Unknown treatment history*	31	

**Table 2:** Comparison of haematological parameters between the cases of malarial infection and controls.

Parameter	Control (n=100) Mean± SD	Cases (n=334) Mean± SD	p value
Haemoglobin (g/dl)	9.23±3.14	10.03±2.77	0.016
TLC (×10^9^/l)	12.10±8.82	8.36±5.43	0.0001
RBC Count (×10^12^/l)	3.65±1.08	3.72±0.99	0.535
MCV (fl)	78.75±11.31	81.21±8.80	0.023
RDW-CV (%)	15.44±2.22	14.93±1.77	0.019
Platelet count (×10^9^/l)	227.38±159.64	86.54±85.36	0.0001
MPV (fl)	7.49±1.31	7.41±2.63	0.767
PDW	9.64±1.30	9.65±1.42	0.947

TLC, Total Leukocyte Count; RBC, Red Blood Cell; MCV, Mean Corpuscular Volume; RDW-CV, Red Cell Distribution Width- Coefficient of Variation; PC, Platelet Count; MPV, Mean Platelet Volume; PDW, Platelet Distribution Width

**Table 3:** Statistical analysis of haematological parameters for diagnosis of malarial infection

Haematological parameter	Cases (n=335)	Control (n=100)	Sensitivity	Specificity	PPV	NPV	LR	95%CI
Hb<12.5(g/dl)	272	85	81.2	15	76.2	19.2	0.9	0.86–1.0
TLC>11(×10^9^/l)	63	44	18.8	56	58.9	17.1	0.4	0.31–0.58
TLC<4(×10^9^/l)	38	10	11.3	90	79.2	23.3	1.1	0.58–2.19
RBC<4(×10^12^/l)	190	63	56.7	37	75.1	20.3	0.9	0.75–1.07
MCV80–100fl	149	47	44.5	53	76	22.2	0.9	0.74–1.20
RDW-CV >15 (%)	140	52	41.8	48	72.9	19.8	0.8	0.64–1.00
PC<150(×10^9^/l)	292	35	87.2	65	89.3	60.2	2.4	1.90–3.26
PDW 6–10	241	67	71.9	33	78.2	26.0	2.0	1.56–2.70
MPV<8fl	130	41	38.8	59	76	22.3	0.9	0.72–1.24
MPV>8fl	205	59	61.5	41	77.7	56.6	1.03	0.86–1.24

PPV, Positive Predictive Value; NPV, Negative Predictive Value; LR, Likelihood Ratio; CI, Confidence Interval; TLC, Total Leukocyte Count; RBC, Red Blood Cell; MCV, Mean Corpuscular Volume; RDW-CV, Red Cell Distribution Width- Coefficient of Variation; PC, Platelet Count; MPV, Mean Platelet Volume; PDW, Platelet Distribution Width

**Table 4:** Statistical analysis of haematological parameters for diagnosis of Plasmodium *vivax* (PV) and Plasmodium *falciparum* (PF) malarial infection.

Parameters	Sensitivity (%)	Specificity (%)	PPV	NPV	LR	95%CI
*TLC<4*×*10^9^/L*						
PV	14.6	90	77.3	31.1	1.45	0.75–2.8
PF	4.3	90	28.6	50.6	0.43	0.14–1.33
*PC<150*×*10^9^/L*						
PV	88	65	85.4	69.9	2.51	1.91–3.29
PF	84.8	65	69	82.3	2.42	1.82–3.20
*PDW 6–10*						
PV	69.5	33	70.7	31.7	1.03	0.88–1.22
PF	82.6	33	53.1	67.3	1.23	1.04–1.45
*MPV>8fl*						
PV	70.8	41	70.8	41	1.2	0.99–1.44
PF	50.4	41	50.4	41	1.10	0.88–1.22

PPV, Positive Predictive Value; NPV, Negative Predictive Value; LR, Likelihood Ratio; CI, Confidence Interval; TLC, Total Leukocyte Count; PC, Platelet Count; MPV, Mean Platelet Volume; PDW, Platelet Distribution Width
